# Mutation K42R in Ribosomal Protein S12 Does Not Affect Susceptibility of *Mycobacterium smegmatis* 16S rRNA A-Site Mutants to 2-Deoxystreptamines

**DOI:** 10.1371/journal.pone.0011960

**Published:** 2010-08-05

**Authors:** Sarath K. Kalapala, Sven N. Hobbie, Erik C. Böttger, Dmitry Shcherbakov

**Affiliations:** 1 Institut für Medizinische Mikrobiologie, Universität Zürich, Zürich, Switzerland; 2 Singapore-MIT Alliance for Research and Technology (SMART), Centre for Life Sciences, Singapore, Singapore; University of Delhi, India

## Abstract

Recent studies have suggested that ribosomal protein S12 modulates 16S rRNA function and susceptibility to 2-deoxystreptamine aminoglycosides. To study whether the non-restrictive K42R mutation in RpsL affects 2-deoxystreptamine susceptibility in *Mycobacterium smegmatis*, we studied the drug susceptibility pattern of various mutants with genetic alterations in the 16S rRNA decoding A-site in the context of wild-type and mutant protein S12. RpsL K42R substitution was found not to affect the drug resistance pattern associated with mutational alterations in 16S rRNA H44.

## Introduction

Ribosomal protein S12 is a critical component of the A-site of the 30S ribosomal subunit and is involved in both tRNA selection and resistance to streptomycin [1], [2]. Mutations in *rpsL* coding for ribosomal protein S12 are known to affect ribosomal accuracy to various extents, resulting in what is characterized as error-restrictive or non-restrictive S12 alterations [3]. Streptomycin inhibits protein synthesis and makes ribosomes error prone by affecting initial tRNA selection and proofreading [4]. Mutations in S12 confer streptomycin resistance by preventing streptomycin binding and/or conferring ribosomal hyperaccuracy; a strongly hyperaccurate phenotype may even manifest as streptomycin-dependence [2]. Various substitutions at positions 42 and 87 are associated with streptomycin resistance. In particular, mutations Lys42 → Arg, Ala or Thr promote high levels of streptomycin resistance; among those Lys42Arg has a non-restrictive phenotype [3], whereas Lys42Ala and Lys42Thr are strongly error-restrictive [3], [5], [6]. S12 substitutions Lys87 → Gln or Gly confer different degrees of streptomycin resistance [7], [8]. The homologous mammalian ribosomal proteins carry Gln-87 (human mitochondrial ribosomes) or Gly-87 (human cytoplasmic ribosomes) in part accounting specificity of drugs for prokaryotic ribosome [7].

More recently it has been suggested that ribosomal protein S12 modulates 16S rRNA function and susceptibility to 2-deoxystreptamine aminoglycosides [5], [6]. 2-deoxystreptamine aminoglycosides are composed of a common core, termed neamine, in which position 4 of a 2-deoxystreptamine ring (ring II) is attached to a glycopyranosyl ring (ring I). Additional sugars are attached to position 5 or 6 of the 2-deoxystreptamine moiety to give rise to 4,5- or 4,6- aminoglycosides (see Supplementary Fig. S1). In 4,5- aminoglycosides, the core is further substituted by one (ribostamycin), two (neomycin, paromomycin) or three (lividomycin) additional sugars attached to position 5 of ring II, whereas in 4,6- aminoglycosides (gentamicin, tobramycin, kanamycin, etc.) the core is further substituted by one additional sugar attached to position 6 of ring II. The drug binding pocket for these compounds consists of an internal loop of 16S rRNA helix 44 – the decoding A-site of the ribosome [9]. We have previously performed extensive genetic studies of 16S rRNA helix 44 in *Mycobacterium smegmatis* to address the role of individual rRNA residues in drug binding (reviewed in [10]). These studies have been conducted mainly in the genetic background of a non-restrictive K42R mutation in ribosomal protein S12 [10]–[16]. The K42R mutation confers high-level resistance to streptomycin and was used as counter-selectable marker in strain construction [15]. The recent reports on the interplay of S12 on 16S rRNA function and susceptibility to 2-deoxystreptamine aminoglycosides prompted us to study in detail the role, if any, between RpsL K42R and mutational alterations in 16S rRNA helix 44 conferring resistance to 2-deoxystreptamines. We find that the non-restrictive RpsL K42R mutation does not affect the 2-deoxystreptamine susceptibility of various rRNA mutations in *M. smegmatis* H44.

## Results and Discussion

### Generation of *M. smegmatis* strains with mutations in S12 and 16S rRNA

*M. smegmatis* was the first eubacterial model organism that was made single rRNA allelic by means of deletion mutagenesis and that allowed for genetic studies of its ribosomal RNA [16]. As a gram-positive mesophilic bacterium, *M. smegmatis* is susceptible to a number of ribosomal antibiotics and sets itself apart from other model organisms by being a close representative of clinically relevant pathogenic bacteria. Early genetic studies on susceptibility to 2-deoxystreptamine aminoglycoside antibiotics relied on *M. smegmatis* strains with a K42R mutation in ribosomal protein S12 [10]–[16]. To substantiate the results and conclusions drawn from these early studies, we constructed a comprehensive set of *M. smegmatis* rRNA mutants in the context of both a wild-type and a mutant S12 (Table S1). The set of 16S rRNA mutants includes alterations of positions 1408 (A1408G), 1491 (G1491C; G1491U; G1491A) and 1409 (C1409U; C1409G).

### Aminoglycoside susceptibility of *M. smegmatis* mutants

Aminoglycoside susceptibility of the mutant strains was determined by minimal inhibitory concentration (MIC) assays. Representatives for each of the two disubstituted 2-deoxystreptamine subclasses were included: the 4,5-disubstituted 2-deoxystreptamines paromomycin and neomycin and the 4,6-disubstituted 2-deoxystreptamines gentamicin, tobramycin and kanamycin. The results are presented in Table 1.

**Table 1:** Drug susceptibility of *M. smegmatis* 16S rRNA mutants: wild-type S12 versus K42R.

16S rRNA residues		S12	MIC (µg/mL)					Reference
1408	1409–1491		Pm	Nm	Gm	Tb	Km	
A	C≡G	wt	1	0.5	1	1	0.5–1	this study
A	C≡G	K42R	1	1	1	1	0.5–1	[13]
G	C≡G	wt	64	>1024	>1024	>1024	>1024	this study
G	C≡G	K42R	64	≥1024	>1024	>1024	>1024	[12]
A	C · C	wt	512	16	16–32	16	16–32	this study
A	C · C	K42R	512	16–32	16–32	16–32	16–32	[12]
A	C · U	wt	512–1024	8–16	32–64	64	64–128	this study
A	C · U	K42R	512	8–16	32	32–64	128	[12]
A	C · A	wt	32–64	2	2	2	1–2	this study
A	C · A	K42R	32	4	2	4	2	[12]
A	U · G	wt	4–8	0.5–1	8	8–16	8–16	this study
A	U · G	K42R	4–8	1	8–16	8–16	16	[12]
A	G · G	wt	32–64	4–8	1–2	8–16	32–64	this study
A	G · G	K42R	16–32	4	2–4	8–16	16–32	[12]

Pm, paromomycin; Nm, neomycin; Gm, gentamicin; Tb, tobramycin; Km, kanamycin A.

**Figure 1:**
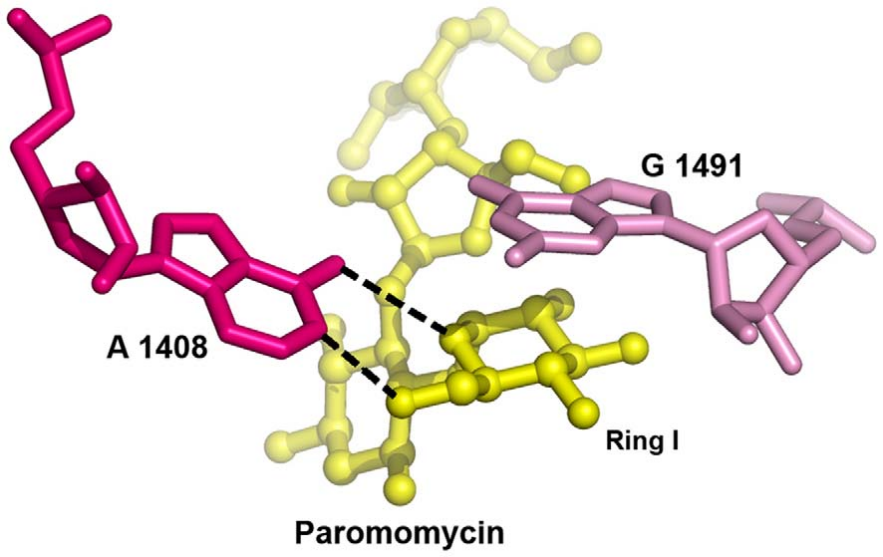
Stacking interaction of ring I with G1491 and pseudo-base pairing of ring I with A1408. The hydrogen bonding contacts between ring I and A1408 are indicated by black broken lines.

Among all A-site mutations that confer aminoglycoside resistance, the A1408G mutation is the most significant. In *M. smegmatis*, the A1408G mutation confers moderate resistance to paromomycin (6′ OH) but high level resistance to 2-deoxystreptamine aminoglycosides with an amino group at the 6′ position of ring I. A key element in drug binding is the pseudo base-pair interaction between the ring of the aminoglycosides I and A 1408 [13], [17], [18]. In case of an adenine, the oxygen of ring I accepts a hydrogen bond from the N6 of A1408, and the amino or hydroxyl group at position 6′ donates a hydrogen bond to the N1 of adenine, accounting for two direct hydrogen bonds between ring I and A1408 (Fig. 1). In case of a 1408 guanine, the 6′ amino group of ring I can no longer form an H bond with the Watson-Crick edge of residue 1408. Additionally, the positive charge of the 6′ amino group creates repulsion against the N1 and N2 amino groups of guanine. As a consequence, the A to G mutation prevents aminoglycoside binding by precluding the proper insertion of ring I into the binding site. In contrast, a 6′- hydroxyl group, as in paromomycin could still become an acceptor of an H bond from N1 or N2 of the guanine, although the resulting pseudo base pair does not appear to promote optimal insertion of ring I, as indicated by decreased ribosomal drug susceptibility.

The Watson–Crick base pair C1409–G1491 forms the base of the drug binding pocket. In the crystal structures [17], [18], G1491 provides a stacking interaction with ring I of the aminoglycosides, thereby stabilizing the pseudo base-pair interaction of ring I with A1408 (Fig. 1). Among all mutations investigated affecting base-pair interaction C1409-G1491, the transversion mutations G1491C and G1491U (resulting in pyrimidine-pyrimidine oppositions) confer the highest level of resistance, in particular to paromomycin. Presumably, a pyrimidine-pyrimidine opposition provides a conformation that sterically hinders the correct positioning of ring I. A pyrimidine-purine opposition is retained following transition of G1491 to A resulting in C1409–1491A. This mutational alteration apparently interferes less with drug binding, as indicated by the drug susceptibility pattern of the mutants. The C1409U mutant shows little resistance to aminoglycosides. Nucleotide C1409 is not involved directly in drug binding, but is responsible for the correct orientation of nucleotide 1491, as 1409 and 1491 form a Watson–Crick base pair. The mutant wobble-base pair interaction 1409U–G1491 is likely to show conformational characteristics resembling those of the wild-type C–G. Mutation C1409G leads to a purine–purine opposition 1409G–G1491 in which the exact nature of interaction is difficult to predict. Surprisingly, the resistance levels conferred by this mutation are low to moderate. In general, sequence alterations in C1409-G1491 while mostly affecting the 6′ OH paromomycin do not discriminate between 4,5- and 4,6-aminoglycosides [10]. This is in agreement with the structural observation that ring I binds in the same orientation irrespective of the substituents at the 2-deoxystreptamine ring [18].

Strains with mutations in 16S rRNA residues 1408, 1409 and 1491 show a mutation-specific drug susceptibility pattern that is independent of the amino acid residue 42 in ribosomal protein S12 (wt *vs* K42R, see Table 1) and that corresponded to previously published data [12]. These results demonstrate that the K42R mutation in ribosomal protein S12 does not affect the susceptibility of *M. smegmatis* H44 mutants to 2-deoxystreptamines. Notably, this finding is not fully congruent with a previous finding in *E. coli* where K42R (the *rpsL*226 allele) reportedly modulated the level of paromomycin resistance of a G1491U mutant [19].

**Figure 2:**
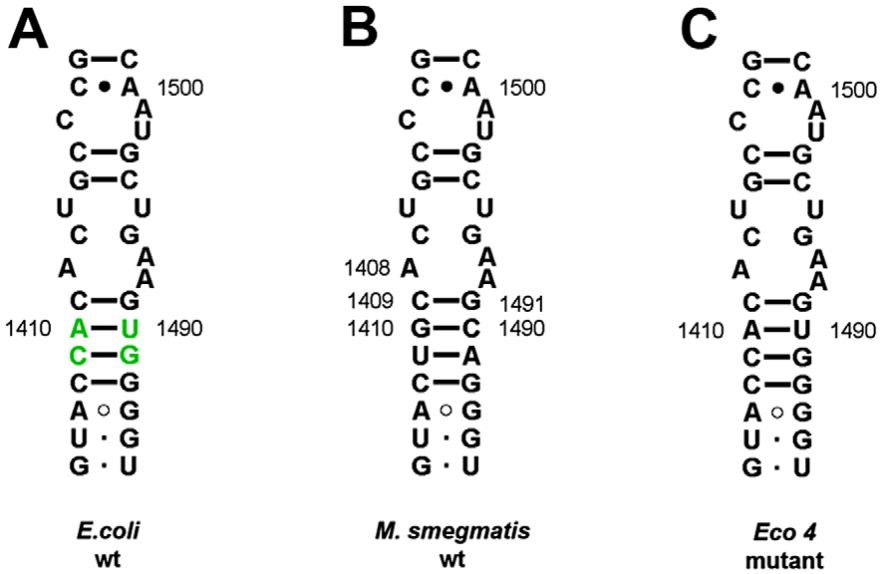
Secondary structure of 16S rRNA helix 44 in the ribosomal decoding site. (**A**) Decoding site of *E. coli* wild-type (Proteobacteria). Four nucleotide positions depicted in green represent residues that are specific for *E. coli* 16S rRNA. (**B**) Decoding site of *M. smegmatis* wild-type (Mycobacteria). (**C**) Decoding site of *M. smegmatis* mutagenised to correspond to the polymorphism observed in *E. coli* i.e., Eco4.

To study whether the different findings reported for *E. coli* are due to phylogenetic differences in the decoding-site rRNA, we constructed *M. smegmatis* with a proteobacteria-like helix 44 (Fig. 2). *M. smegmatis* and *E. coli* differ in 16S rRNA residues 1410–1490 and 1411–1489: 1410–1490 G-C (*M. smegmatis*) *versus* A-U (*E. coli*), 1411–1489 U-A (*M. smegmatis*) *versus* C-G (*E. coli*). We found that the various H44 mutations (A1408G, G1491A, G1491C, G1491U) resulted in identical drug susceptibility patterns regardless of a mycobacterial and proteobacterial H44 sequence context at residues 1410–1490 and 1411–1489 (data not shown).

### Structural analysis of K42 mutations

**Figure 3:**
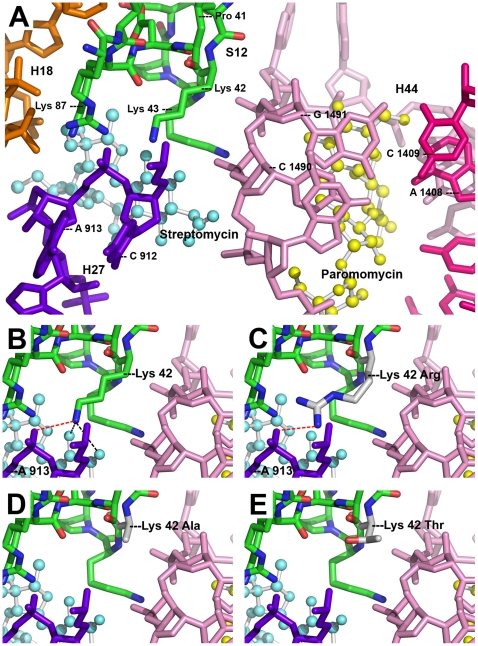
Three-dimensional crystal structure of *T. thermophilus* ribosomal decoding A-site with bound paromomycin and streptomycin. (**A**) General view of the A-site. Amino-acid residues of S12 (green) are shown labelled according to atoms: carbon – green, nitrogen – blue, oxygen – red. 16S rRNA helices are indicated as follows: H44 strand I (pink), H44 strand II (magenta), H27 (violet), H18 (orange). Streptomycin (light blue) and paromomycin (yellow). (**B**) Close up of wild type K42. Hydrogen bonds to streptomycin (black dotted lines) and salt bridge to A913 (red dotted line) are shown. (**C**) Close up of mutant K42R. Salt bridge (red dotted line) is shown. (**D**) Close up of mutant K42A. (**E**) Close up of mutant K42T. (Protein Data Bank, 1FJG.pdb).

Crystal structures of streptomycin bound to the small ribosomal subunit of *T. thermophilus* [17] have revealed two direct hydrogen bonds between streptomycin and the lysine residue 42 of ribosomal protein S12 (Fig. 3A, 3B). K42 forms an additional contact to the phosphate backbone of 16S rRNA helix 27 (H27) via a salt bridge to the phosphate group of residue A913. Superimposition of the K42R substitution disrupts the hydrogen bonding to streptomycin (Fig. 3C), accounting for the streptomycin resistance of K42R mutants. However, amino-acid substitution K42R leaves the salt bridge to H27 intact (Fig. 3C). Thus, the general structure of the A-site remains intact and rate and fidelity of translation remains unaffected [17]. This is in agreement with the observation that K42R is the only known mutation in S12 that confers streptomycin resistance but at the same time does not result in a hyper-accurate (i.e. restrictive) phenotype [3].

In contrast to K42R, mutations K42A or K42T disrupt the salt bridge to H27 (Fig. 3D, 3E). This mostly accounts for the restrictive phenotype of these mutations [17]. While K42A does not interfere with the binding of paromomycin directly [5], the hyperaccurate phenotype of the variant K42A ribosome in part functionally antagonizes aminoglycoside-induced misreading. Thus, these ribosomes show paromomycin-induced misreading only at much higher drug concentrations compared to wild-type [5].

### Conclusions

Our data demonstrate that K42R in ribosomal protein S12 does not affect resistance to 2-deoxystreptamine aminoglycosides as conferred by 16S rRNA mutations in H44, and that S12 most likely plays little role in the species-specific pattern of susceptibility to 2-deoxystreptamines. This conclusion is supported by the observation that mutations G1645A/A1754G in *S. cerevisiae* 18S rRNA (homologous to *E. coli* residue A1408/G1491) reversed the natural resistance of yeast cytoplasmic ribosomes, i.e, increased the susceptibility to aminoglycosides from completely resistant (MIC>5000 µg/ml) up to highly susceptible (MIC about 3 µg/ml). These levels of drug susceptibility are similar to those found in drug susceptible *E. coli* (MIC about 2.5–5 µg/ml) [20]. Thus, irrespective of ribosomal protein S12 (*S. cerevisiae* or *E. coli*), susceptibility to 2-deoxystreptamines is apparently determined by the nucleotide residues in drug binding pocket of 16S rRNA. This finding contrasts with the view of a universal interrelation between S12, base pair 1409–1491 and 2-deoxystreptamine susceptibility. Instead, it suggests that functional interactions between S12 and the 2-deoxystreptamine binding site are limited to certain bacterial genera and/or specific *rpsL* mutations. Interaction of S12 and 2-deoxystreptamine aminoglycosides apparently is not at the level of drug binding, but provoked by interference of two opposite effects on translation fidelity induced by error-restrictive *rpsL* mutations and misreading-inducing aminoglycoside antibiotics. As a result, this interplay is limited to S12 amino acid substitutions which confer a hyperaccurate phenotype and thus are functional antagonists of drug-induced misreading.

## Materials and Methods

### Strains used in this study

A single rRNA allelic strain *M. smegmatis* Δ*rrnB* (SZ380) was generated by unmarked deletion mutagenesis of the *rrnB* operon in *M. smegmatis* mc^2^155. A suicide vector pH022 containing two DNA fragments flanking the *rrnB* operon (generated by PCR), a selectable marker (Gm^R^) and counter-selectable marker *sacB* (both outside the *rrnB* DNA fragments), was transformed into *M. smegmatis* mc^2^155. A single rRNA allelic derivative was obtained by a two-step selection procedure: selection of transformants on agar plates containing gentamicin followed by a counter-selection step on agar plates with sucrose. Deletion of *rrnB* was confirmed by Southern blot analysis and partial sequencing.

Mutagenesis of the H44 decoding-site RNA was performed in strain *M. smegmatis* Δ*rrnB* (SZ380) and in strain *M. smegmatis* Δ*rrnB rpsL* K42R (SZ004) [12]. rRNA mutations were generated by PCR, cloned into vector pMIH-rrnB and introduced into the 16S rRNA A-site of the single rRNA allelic strain SZ0380 by RecA-mediated homologous recombination as described [12]. For a list of strains and plasmids see Tables S1 and S2 in Supplementary Data.

### Minimal inhibitory concentration (MIC) assay

Drug susceptibility was studied by determining Minimal Inhibitory Concentrations (MIC). MIC tests were performed in a microtiter plate format as described [12]. In brief, freshly grown *M. smegmatis* cultures were resuspended in LB broth supplemented with 0.05% of Tween 80, diluted to an absorbance at 600 nm of 0.025 and incubated in the presence of 2-fold serial dilutions of 2-deoxystreptamine aminoglycosides. After incubation at 37°C for 72 h, the MIC was recorded as the lowest concentration of drug inhibiting visible growth.

### Structural modelling

PyMol (DeLano Scientific) was used to render the structure of the A-site of 30S ribosomal subunit from *T. thermophilus* (Protein Data Bank, 1FJG.pdb) [21].

## Supporting Information

Figure S1 Chemical structures of disubstituted 2-deoxystreptamine antibiotics used in this study.
(0.48 MB DOC)

Table S1 Strains used in this study.
(0.04 MB DOC)

Table S2 Plasmids used in this study.
(0.03 MB DOC)
