# Supplementary material for: Mutation K42R in Ribosomal Protein S12 Does Not Affect Susceptibility of Mycobacterium smegmatis 16S rRNA A-Site Mutants to 2-Deoxystreptamines
Source: PLoS One. 2010 Aug 5;5(8):e11960. doi: 10.1371/journal.pone.0011960 (PMC2916820; doi:10.1371/journal.pone.0011960)
Supplement: Figure S1 — Chemical structures of disubstituted 2-deoxystreptamine antibiotics used in this study. (0.48 MB DOC) [file pone.0011960.s001.doc]

**Figure S1. Chemical structures of disubstituted 2-deoxystreptamine antibiotics used in this study.**

**
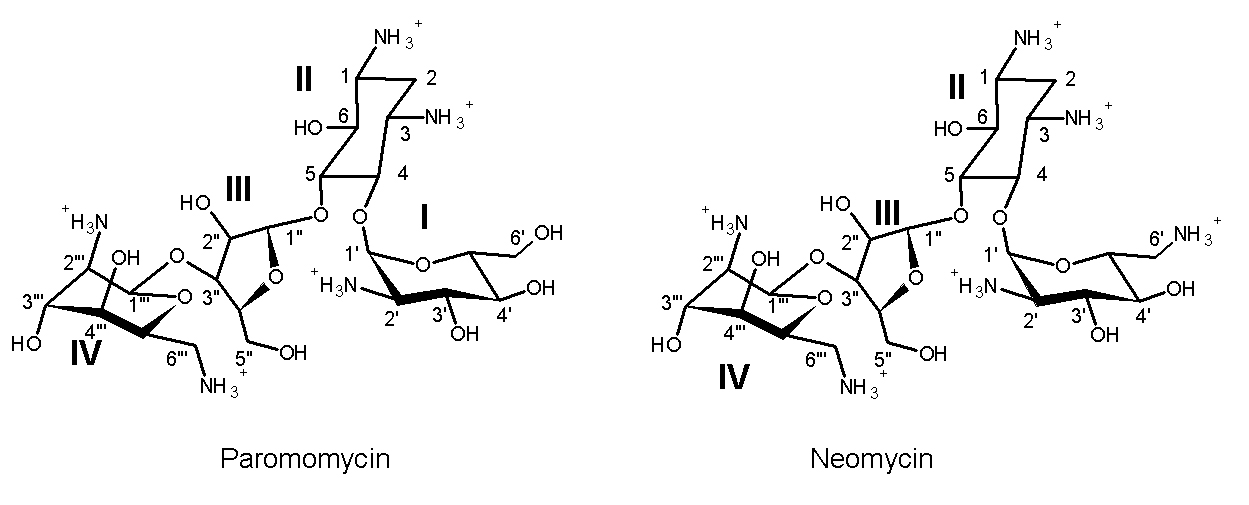
**

**
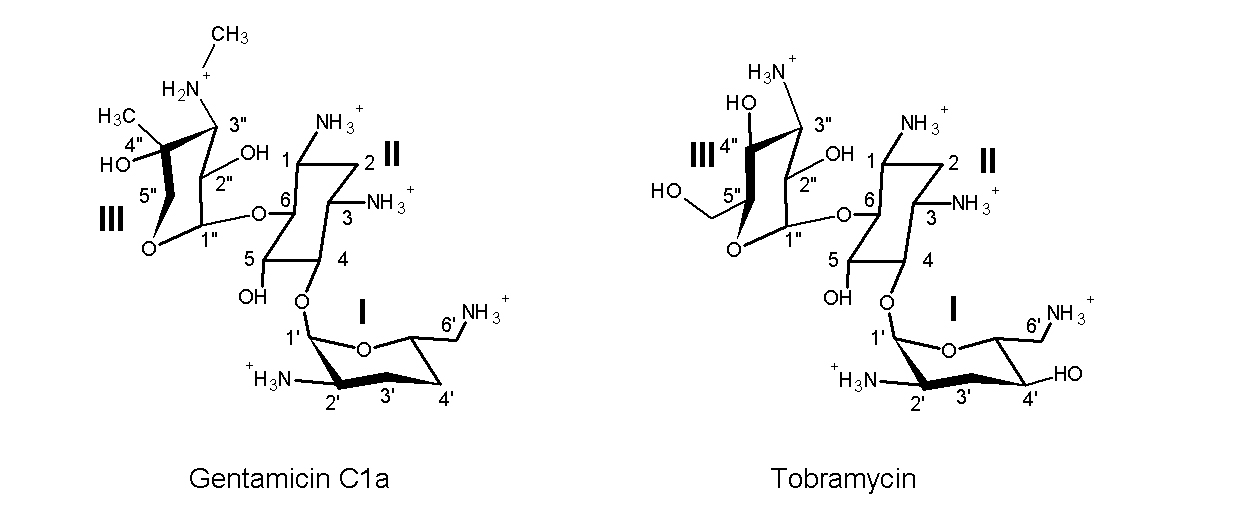
**

**
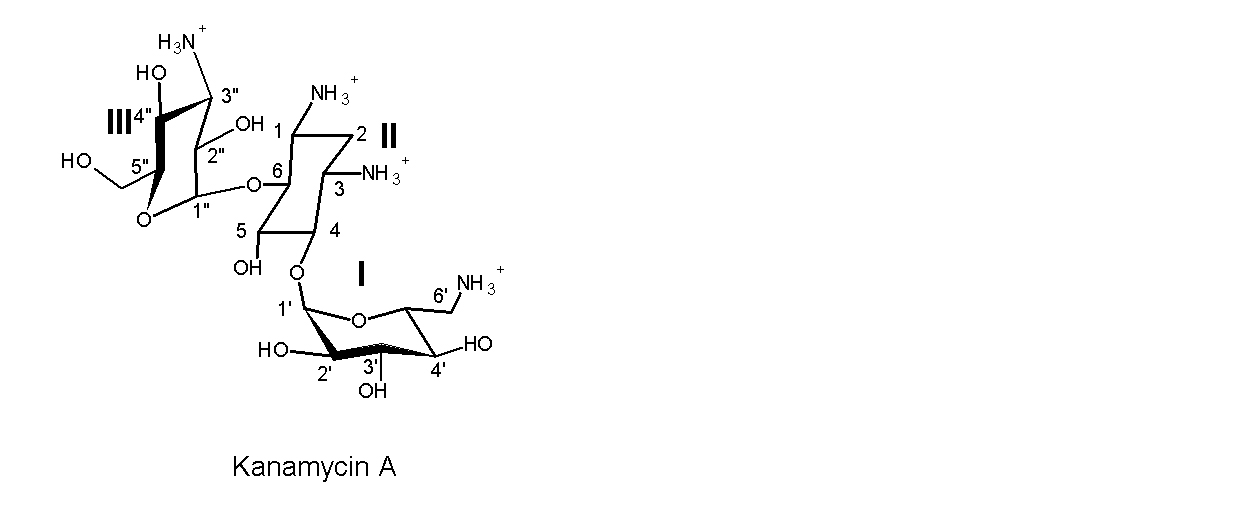
**
