# Supplementary material for: Mutation K42R in Ribosomal Protein S12 Does Not Affect Susceptibility of Mycobacterium smegmatis 16S rRNA A-Site Mutants to 2-Deoxystreptamines
Source: PLoS One. 2010 Aug 5;5(8):e11960. doi: 10.1371/journal.pone.0011960 (PMC2916820; doi:10.1371/journal.pone.0011960)
Supplement: Table S1 — Strains used in this study. (0.04 MB DOC) [file pone.0011960.s002.doc]

**Table S1**. Strains used in this study.

| ***M. smegmatis* strain** | **Parental strain** | ***rpsL*** | **16S rRNA mutation** | **Mutagenesis** | **Reference** |
| --- | --- | --- | --- | --- | --- |
|  |  |  |  |  |  |
| SZ380 ∆*rrnB* *rrnA*^+^ | mc^2^155 | wt | wt | Recomb. | this study |
| SZ004 ∆*rrnB* *rrnA*^+^ | mc^2^155 SMR5 | K42R | wt | Recomb. | [13] |
| ∆*rrnB* *rrnA*(G1491A) | SZ380 | wt | G1491A | Recomb. | this study |
| ∆*rrnB* *rrnA*(G1491A) | SZ004 | K42R | G1491A | Recomb. | [12] |
| ∆*rrnB* *rrnA*(G1491C) | SZ380 | wt | G1491C | Recomb. | this study |
| ∆*rrnB* *rrnA*(G1491C) | SZ004 | K42R | G1491C | Recomb. | [12] |
| ∆*rrnB* *rrnA*(G1491U) | SZ380 | wt | G1491U | Recomb. | this study |
| ∆*rrnB* *rrnA*(G1491U) | SZ004 | K42R | G1491U | Recomb. | [12] |
| ∆*rrnB* *rrnA*(C1409G) | SZ380 | wt | C1409G | Recomb. | this study |
| ∆*rrnB* *rrnA*(C1409G) | SZ004 | K42R | C1409G | Recomb. | [12] |
| ∆*rrnB* *rrnA*(C1409U) | SZ380 | wt | C1409U | Recomb. | this study |
| ∆*rrnB* *rrnA*(C1409U) | SZ004 | K42R | C1409U | Recomb. | [12] |
| ∆*rrnB* *rrnA*(A1408G) | SZ380 | wt | A1408G | Recomb. | this study |
| ∆*rrnB* *rrnA*(A1408G) | SZ004 | K42R | A1408G | Recomb. | [12] |
|  |  |  |  |  |  |

Recomb., mutagenesis by RecA-mediated gene conversion.
