# Supplementary material for: Mutation K42R in Ribosomal Protein S12 Does Not Affect Susceptibility of Mycobacterium smegmatis 16S rRNA A-Site Mutants to 2-Deoxystreptamines
Source: PLoS One. 2010 Aug 5;5(8):e11960. doi: 10.1371/journal.pone.0011960 (PMC2916820; doi:10.1371/journal.pone.0011960)
Supplement: Table S2 — Plasmids used in this study. (0.03 MB DOC) [file pone.0011960.s003.doc]

**Table S2**. Plasmids used in this study.

| **Number** | **Plasmid** | **Marker** | **16S rRNA mutation** | **Reference** |
| --- | --- | --- | --- | --- |
| pH022 | pGEM7-*rrnB5’3’-sacB-aph* | Gm | - | this study |
| PZ176 | pMV361∆*aph*-*hyg*-1491A | Hyg | G1491A | [12] |
| PZ178 | pMV361∆*aph*-*hyg*-1491C | Hyg | G1491C | [12] |
| PZ177 | pMV361∆*aph*-*hyg*-1491U | Hyg | G1491U | [12] |
| PZ191 | pMV361∆*aph*-*hyg*-1409G | Hyg | C1409G | [12] |
| PZ175 | pMV361∆*aph*-*hyg*-1409U | Hyg | C1409U | [12] |
| pH128 | pMV361∆*aph*-*hyg*-1408G | Hyg | A1408G | [13] |
|  |  |  |  |  |

Hyg, hygromycin; Gm, gentamicin.
